# Dual Networks for High-Precision and High-Speed Registration of Brain Electron Microscopy Images

**DOI:** 10.3390/brainsci10020086

**Published:** 2020-02-07

**Authors:** Chang Shu, Tong Xin, Fangxu Zhou, Xi Chen, Hua Han

**Affiliations:** 1 Institute of Automation, Chinese Academy of Sciences, Beijing 100190, China; shuchang2015@ia.ac.cn (C.S.); xintong2018@ia.ac.cn (T.X.); zhoufangxu2017@ia.ac.cn (F.Z.); 2 School of Artificial Intelligence, University of Chinese Academy of Sciences, Beijing 100049, China; 3 School of Future Technology, University of Chinese Academy of Sciences, Beijing 100049, China; 4 The Center for Excellence in Brain Science and Intelligence Technology, CAS, Shanghai 200031, China; 5 National Laboratory of Pattern Recognition, CASIA, Beijing 100190, China

**Keywords:** computer vision, image processing, deep learning, image registration, electron microscopy image, dual network architecture, unsupervised learning

## Abstract

It remains a mystery as to how neurons are connected and thereby enable use to think, and volume reconstruction from series of microscopy sections of brains is a vital technique in determining this connectivity. Image registration is a key component; the aim of image registration is to estimate the deformation field between two images. Current methods choose to directly regress the deformation field; however, this task is very challenging. It is common to trade off computational complexity with precision when designing complex models for deformation field estimation. This approach is very inefficient, leading to a long inference time. In this paper, we suggest that complex models are not necessary and solve this dilemma by proposing a dual-network architecture. We divide the deformation field prediction problem into two relatively simple subproblems and solve each of them on one branch of the proposed dual network. The two subproblems have completely opposite properties, and we fully utilize these properties to simplify the design of the dual network. These simple architectures enable high-speed image registration. The two branches are able to work together and make up for each other’s drawbacks, and no loss of accuracy occurs even when simple architectures are involved. Furthermore, we introduce a series of loss functions to enable the joint training of the two networks in an unsupervised manner without introducing costly manual annotations. The experimental results reveal that our method outperforms state-of-the-art methods in fly brain electron microscopy image registration tasks, and further ablation studies enable us to obtain a comprehensive understanding of each component of our network.

## 1. Introduction

Image registration is a long-standing problem in computer vision and medical image processing. The aim of image registration is to estimate the deformation field between two images and warp source images to reference images according to the estimated deformation field. This task has wide applicability in neuroscience. To obtain a comprehensive understanding of the circuitry mechanisms underlying brain neuron structures, mechanically cut sections are imaged by electron microscopy. Due to the large deformation and artefacts introduced in the data acquisition step, a registration method that can recover the connectivity among neurons is of great importance.

Current learning-based methods generally directly regress the deformation field from two coarsely aligned images. Coarse alignment is usually conducted by off-the-shield software, and high alignment quality is not guaranteed. The whole process is not end to end and cannot fully exploit the advantages of deep learning. Most methods pursue the high-precision prediction of the deformation field at the expense of computational complexity. Many well-designed modules are progressively added to the networks, causing a decrease in inference speed.

In this work, we suggest that a complex architecture is not necessary, and we simply need to reformulate the original problem. We split the problem into two parts: estimating a linear deformation and estimating the remaining nonlinear deformation, which can be considered as a residual of the previous linear deformation.

The linear deformation can be parameterized by an affine transformation. A simple architecture is sufficient to regress its six parameters. Since linear deformations pay greater attention to the global transformation of the image, they do not focus on local details. Utilizing this property, we can use low-resolution inputs to reduce the computational complexity. After the linear deformation has been estimated, we warp the original images to eliminate this part of the deformation. The remaining nonlinear deformation is estimated from these linearly warped images. Since a large part of the deformation represented by the affine transformation has been eliminated, the remaining nonlinear deformation can be seen as small variations of fine details; such deformation focuses on local structures, no high-level semantic information is involved, and a shallow neural network is sufficient.

To summarize, we decompose the original problem to reduce the difficulty in solving it. The two subproblems have completely opposite properties, and we utilize these properties to simplify the design of the corresponding estimation branches of the overall network, resulting in a simple architecture. The two branches are combined in a dual manner and make up for each other’s drawbacks. The resulting dual-network architecture is able to perform image registration with high precision and high speed.

Furthermore, to eliminate the cost of manual annotation, we propose a training scheme for unsupervised image registration. No knowledge of ground truth is needed, and the registration quality is totally regularized by our specially designed losses.

The experimental results reveal that our method outperforms state-of-the-art methods in the fly brain electron microscopy image registration task, and further ablation studies enable us to obtain a comprehensive understanding of each component of our network.

## 2. Related Work

In recent years, an increasing number of biological brain volumes have been reconstructed from serial-section electron microscopy images, e.g., the adult Drosophila melanogaster brain [1], the larval zebrafish brain [2], the mouse neocortex [3], and layer 4 of the mouse barrel cortex [4]. Due to severe deformation introduced by the data acquisition process, image registration is a key component for eliminating deformation among serial sections and recovering the continuity of the whole brain. Several mature image registration algorithms have been proposed and made public.

### 2.1. Traditional Registration Methods

All traditional registration methods calculate a parameterized deformation field based on the matches of sparse features such as SIFT [5], SURF [6] and BRISK [7]. LeastSquare [8] performs registration based on SIFT [5] correspondences extracted from adjacent sections, and a least square problem is optimized to obtain the final registration results. BUnwarpJ [9] utilizes a nonlinear spatial transformation in the form of cubic B-splines for registration based on coarse registration results (e.g., the result of LeastSquare). CW_R_color [10] aligns neighboring section image pairs via area-based matching and a modified feature extraction method. The elastic method [11] models image registration as a spring-connected particle system, where every pair of correspondences is connected by zero-length springs; releasing all the springs would lead to a nonlinear registration. All these methods are based on iterative optimizations, and it is difficult for them to achieve the efficiency of learning-based methods. Their accuracy is largely affected by the quality of the feature matching; however, these hand-crafted descriptors fail to extract reliable correspondences from complex electron microscopy images.

### 2.2. Learning-Based Methods

With the development of deep learning, deep learning-based methods have gradually been proposed in recent years. As a common way to estimate the deformation field, well-developed optical flow prediction networks [12,13] have been incorporated for image registration. Another technique is to introduce the brilliant ideas from traditional methods to deep learning such as in [14]. ssEMnet [15] proposed an end-to-end architecture by aligning feature maps extracted from input image pairs, and the feature maps are obtained from a pretrained autoencoder. Mitchell et al. [16] leveraged a Siamese CNN for feature maps and aligned the feature maps in a coarse-to-fine manner. Since deformation field regression is a challenging task, to guarantee satisfactory accuracy, complex stacked architectures are utilized, which greatly decrease the prediction speed.

## 3. Dual Network Architecture

### 3.1. Motivation

Our aim is to estimate the high-precision deformation field in minimal time. This task is not trivial since common practices trade off time for accuracy. To this end, we adopt a divide-and-conquer scheme to split a difficult deformation field estimation problem into two relatively simple subproblems. In this way, these two subproblems can be solved by neural networks with simple architectures.

We divide the original deformation into a linear part and a nonlinear part. Although common deformations are nonlinear, we can still extract a linear part therein, and the remaining nonlinear deformation can be treated as a residual. In practice, linear deformations (especially rigid deformations) are in the majority because sections are placed in various poses; this situation is inevitable but will not destroy the morphology of the brains. On the other hand, nonlinear deformation is often caused by sectioning and ruins the original brain structures, causing issues in subsequent analyses. With the development of various techniques, many precautions are being taken to largely avoid this type of artefact. Therefore, instead of directly regressing the deformation field from image pairs, we design a dual-network architecture whose two branches—LinearNet and NonlinearNet—are aimed at handling the linear deformation and the remaining nonlinear deformation, respectively. The combination of the estimated linear deformation and remaining nonlinear deformation forms the final deformation. Our workflow first estimates the linear deformation and then uses the estimated linear deformation to warp the source images to eliminate the majority of the deformation; consequently, the remaining nonlinear deformation is estimated as a residual.

Before utilizing the different characteristics of the linear deformation and nonlinear deformation, we summarize their properties in the following.

**Properties of linear deformations:**

- A linear deformation can be parameterized as a rigid transformation, similarity transformation, affine transformation etc. A small quantity of parameters need to be estimated, and the regression process is relatively simple.
- A linear deformation focuses on the global transformation of the whole image and is less focused on spatial variations of local details.
- A linear deformation pays more attention to high-level semantic features, which plays an important part in global transformation regression. Correspondingly, low-level semantic information becomes less important, and we can avoid unnecessary computations in this part.

**Properties of nonlinear deformations:**

- Although some methods choose to parameterize a nonlinear deformation as thin-plate splines or B-splines to simplify the calculation, these forms can only cover finite types of nonlinear deformations. One flexible method is representing a deformation as a field; however, extra regularization terms, such as smoothness penalties, are needed, and this approach has difficulties regressing large deformations.
- A nonlinear deformation is not inclined to have global consistency. Every pixelwise spatial variation can be different; however, in real-world applications, it is usually locally smooth.
- The regression of nonlinear deformations relies more on low-level semantic features, and high-level semantic information is less useful.

Through analysing the properties of linear and nonlinear deformations, we utilize these properties to guide the design of our dual-network architecture. The design principles of LinearNet and NonlinearNet, which fully explore the respective properties of two deformations, are summarized as follows.

**Design principles of LinearNet:**

- LinearNet is designed to have a simple architecture, i.e., an encoder with sequential standard convolutional layers to regress a six-dimensional vector as the parameters of an affine transformation.
- Only the high-level semantic features will be leveraged to regress the final parameters, and low-level semantic features are merely used to generate high-level semantic features.
- The inputs of the network are resized to low resolution. Since a linear deformation focuses on a global transformation, high resolution is unnecessary.

**Design principles of NonlinearNet:**

- NonlinearNet is designed to be shallow because nonlinear deformations attach minimal importance to high-level semantic information.
- Low-level semantic information is reused via skip connections [17].
- High-resolution images are needed; therefore, spatial variations at fine details will be well distinguished.

Through fully utilizing the respective properties of the two deformations, we are able to simplify the architectures of LinearNet and NonlinearNet as much as possible. Unnecessary computations are abandoned, enabling us to increase the inference speed and retain satisfactory registration accuracy simultaneously. In the following, we will describe the detailed overall pipeline of our dual network.

### 3.2. Overall Pipeline

**Figure 1:**
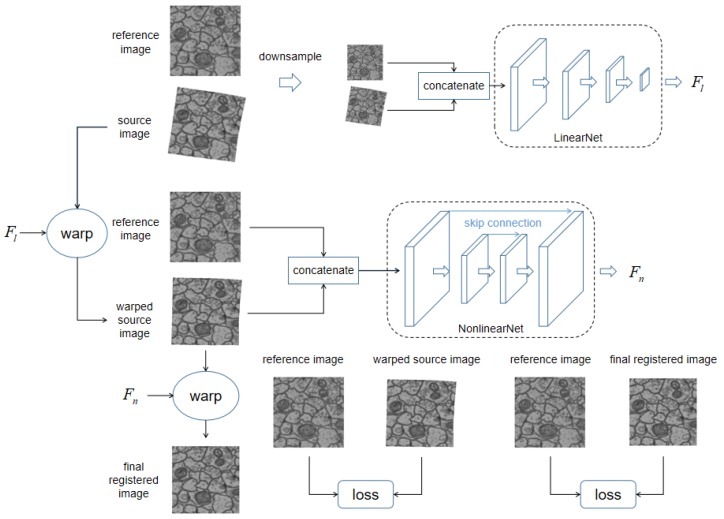
An illustration of our proposed dual network architecture, its aim is to register source image to reference image. Our network includes two parts: LinearNet and NonlinearNet, which respectively regress the linear deformation field $F_{l}$ and the nonlinear deformation field $F_{n}$ between reference image and source image. LinearNet regresses the linear deformation field $F_{l}$ between original image pairs. Linear deformation field $F_{l}$ is then used to eliminated linear deformation within source image by warping, resulting in the warped source image. The warped source image is concatenated with reference image as inputs for NonlinearNet to regress remaining nonlinear deformation field $F_{n}$. Consequently, the nonlinear deformation field $F_{n}$ is used to warp previously warped source image to get final registered image. The difference between reference image and warped source image, and the difference between reference image and warped source image are all measured as training losses.

The overall pipeline of our dual network is illustrated in Figure 1. The network is composed of two subnetworks, LinearNet and NonlinearNet, which aim to estimate the linear deformation field $F_{l}$ and the nonlinear deformation field $F_{n}$, respectively.

The inputs of LinearNet are the low-resolution concatenated source and target images ($I_{s}^{k},I_{t}^{k}$), where *k* refers to the $\frac{1}{k}$ resolution of the original images. LinearNet computes the linear deformation field $F_{l}$, parameterized by six parameters of an affine transformation, and warp the source image according to $F_{l}$, resulting in a warped source image ${\dot{I}}_{s}=W\left(I_{s};F_{l}\right)$, where *W* denotes a warping function that can warp the input image according to the input deformation field. The warping process is fully differentiated and can be implemented as a neural network layer; for detailed information, please refer to STN [18]. The discrepancy $L(I_{t},{\dot{I}}_{s})$ between $I_{t}$ and ${\dot{I}}_{s}$ is evaluated as losses to guide the training of LinearNet. The specific setting of the training losses will be elaborated upon in the next subsection.

Similarly, the inputs of NonlinearNet are high-resolution concatenated previously warped source and target images (${\dot{I}}_{s},I_{t}$). NonlinearNet computes the nonlinear deformation field $F_{n}$ and warps a previous warped source image ${\dot{I}}_{s}$ according to $F_{n}$, resulting in a newly warped source image ${\ddot{I}}_{s}=W\left(I_{s};F_{n}\right)$. The discrepancy $L(I_{t},{\ddot{I}}_{s})$ between $I_{t}$ and ${\ddot{I}}_{s}$ is evaluated as losses to guide the training of NonlinearNet.

### 3.3. Loss

In this section, we report the components of our training loss function. No ground truth is involved throughout the process. Instead of coming from labeled data, the supervisory signal is obtained by minimizing the difference between registered image pairs.

**Image Intensity Error.** A straightforward metric used to evaluate the registration quality is to measure the image intensity difference between registered image pairs. Here, the registered image pairs from two stages are evaluated. This image intensity error is

(1) $$L_{I}=∥I_{t}-{\dot{I}}_{s}{∥}_{1}+{∥I_{t}-{\ddot{I}}_{s}∥}_{1}$$

**Image structural similarity.** Since the image intensity error is only a pixelwise metric, it does not consider the similarity between the structures. To emphasize the alignments of neuron structures, we introduce SSIM [19] to measure the structural similarity of registered image pairs.

(2) $$L_{SSIM}=\frac{1-SSIM(I_{t},{\dot{I}}_{s})}{2}+\frac{1-SSIM(I_{t},{\ddot{I}}_{s})}{2}$$

Since the value of the SSIM metric is between -1 and 1 and since a higher SSIM value is equivalent to a lower discrepancy between two input images, a linear conversion is used. The detailed formulation of the SSIM will be described in Section 4.2.

**The constraint for linear deformation estimation.** Since the affine transformation involves scaling the images, at the beginning of the training, the randomly initialized LinearNet may generate an impractical affine transformation, which will degrade the subsequent optimization. We add an extra constraint to avoid this situation. We require the estimated affine transformation to be close to an identity transformation; in other words, every value of the corresponding linear deformation field must be near zero:

(3) $$L_{lc}={∥F_{l}∥}_{1}$$

**The constraint for nonlinear deformation estimation.** Since our nonlinear deformation is formulated as a field, a fine regularization needs to be adopted to avoid excessive randomness in the estimated field. A common practice is to add a smoothness loss, as described below.

(4) $$L_{nc}=∥\nabla F_{n}{∥}_{1}+{∥\nabla^{2}F_{n}∥}_{1}$$

where ∇ and $\nabla^{2}$ are the first-order and second-order differential operators of matrices. Here, the first-order and second-order smoothness losses are used.

**Total Loss.** The total loss for training the dual network is the combination of the above three losses:

(5) $$L_{total}=\lambda_{1}L_{I}+\lambda_{2}L_{SSIM}+\lambda_{3}L_{lc}+\lambda_{4}L_{nc}$$

### 3.4. Implementation Details

We report the detailed settings of the network architectures and training schemes in this subsection.

**LinearNet.** The input resolution of LinearNet is set to 1/2 of the original resolution (256×256). The output represents the linear deformation, which is parameterized in the form of an affine transformation (six parameters need to be estimated). LinearNet has an encoder architecture, where multiple convolutional layers with a stride of 2 are used to gradually extract high-level semantic information, and a global average pooling layer (GAP) is adopted to obtain the final six-dimensional vector. The detailed network settings are shown in Table 1. To avoid the situation where a bad initialization will cause LinearNet to output an impractical affine transformation, we multiply the vector with 0.01 and then add it to a vector reshaped from an identity transformation. In this way, LinearNet is inclined to output an identity transformation at the beginning of training. To make the network able to output negative values, rectified linear units (ReLUs) [20] are used as the activation function only in the first several layers.

**NonlinearNet.** The goal of NonlinearNet is to estimate the remaining nonlinear deformation after the linear deformation has been eliminated by LinearNet. Some methods tend to model a nonlinear deformation by thin-plate splines or B-splines; however, these representations can only cover a part of the nonlinear deformation, and most nonlinear deformations cannot be parameterized. In this work, we represent the nonlinear deformation as a field; at this time, it is easy to optimize since, in practice, a large proportion of common deformations is a linear deformation.

The input resolution of NonlinearNet is equal to the original resolution (512×512). The output is a two-channel matrix with the same resolution as the input images. NonlinearNet has a shallow encoder-decoder architecture, where multiple convolutional layers and upsampling layers are used to regress the final deformation field. The detailed network settings are shown in Table 2. To ensure that the network is able to generate negative values, LeakyReLU [21] is used as the activation function. To constrain the range of the estimated field to be reasonable, the output of the last layer is activated by the tanh function and multiplied by 0.1 to constrain the value to the range of −0.1 to 0.1. Note that batch normalization (BN) [22] is important for nonlinear deformation regression, and we experimentally found that the introduction of BN will largely accelerate the training process and facilitate convergence.

**Training settings.** Our models are implemented on PyTorch [23] and trained for 20 epochs using the Adam [24] optimizer, with a batch size of 2, on 1 Titan Xp GPU. The learning rate is set to 0.001 and halved twice at the 10th epoch. The weights in the total loss (Equation (5)) are set as $\lambda_{1}=0.15,\;\lambda_{2}=0.85,\;\lambda_{3}=1,\;\lambda_{4}=0.1$.

## 4. Experiments

### 4.1. Data

The dataset we use for the evaluation is named CREMI and consists of 5 um^3^ volumes of serial section EM of the adult fly brain. The dataset contains 125 section images with an image resolution of 1250×1250 pixels. It has been manually annotated and registered, and it can be treated as ground truth. Following standard practice as described in [15,25], a random deformation is applied to each image and its label to mimic real-world data in the form of an affine transformation along with a thin plate spline transformation. The affine transformation is controlled by random vectors, and the thin plate spline deformation is controlled by random vectors on random positions, where the random vectors are sampled from a normal distribution with zero mean and the random positions are uniformly distributed over the image grid. This process expands the original dataset to 1240 image pairs, and then, we use a 0.8/0.1/0.1 split for for training, validation and testing, respectively.

### 4.2. Performance Metrics

**SSIM.** As a metric to evaluate the similarity between two images, the SSIM can be used as not only a training loss function but also a testing measurement. This measures similarity in three aspects: illuminance, contrast and structure. We adopt this metric to measure the similarity between registered image pairs, as shown in Table 3.

(6) $$SSIM\left(I_{1},I_{2}\right)=\frac{2u_{1}u_{2}+C_{1}}{u_{1}^{2}+u_{2}^{2}+C_{1}}\cdot \frac{2\sigma_{1}\sigma_{2}+C_{2}}{\sigma_{1}^{2}+\sigma_{2}^{2}+C_{2}}\cdot \frac{\sigma_{12}+C_{3}}{\sigma_{1}\sigma_{2}+C_{3}}$$

where $u_{i}$ refers to the mean intensity of the i-th image, $\sigma_{i}$ refers to the unbiased standard deviation of the i-th image, $\sigma_{ij}$ refers to the correlation coefficient between the i-th image and the j-th image, and $C_{1}$, $C_{2}$, and C3 are three small constants used to avoid infinite values. Here, the mean intensity, unbiased standard deviation and similarity between normalized images (i.e., correlation coefficient) are used to represent the illuminance, image contrast and structural similarity, respectively. It is a common practice to divide the original images into patches and evaluate the SSIM over each patch. A smaller patch size will lead to finer measurements. The patch size is often set to 3×3, which is also used in our work.

**Dice.** As a standard medical image registration quality metric, Dice is widely used. Since neurons in the CREMI dataset have been annotated, we can adopt the dice metric to evaluate the registration quality. Specifically, we register the electron microscopy images along with their corresponding labeled images, and then, we evaluate the overlap of neurons between the registered labeled images and the ground truth. This is mathematically formulated as

(7) $$Dice\left(A,B\right)=2*\frac{\left|A\cap B\right|}{\left|A|+|B\right|}$$

where *A* and *B* refer to two neurons in the warped labeled images and ground truth, respectively. As a common practice, we choose the 50 largest neurons and calculate the average Dice metric.

### 4.3. Comparison with the State of the Art

**Figure 2:**
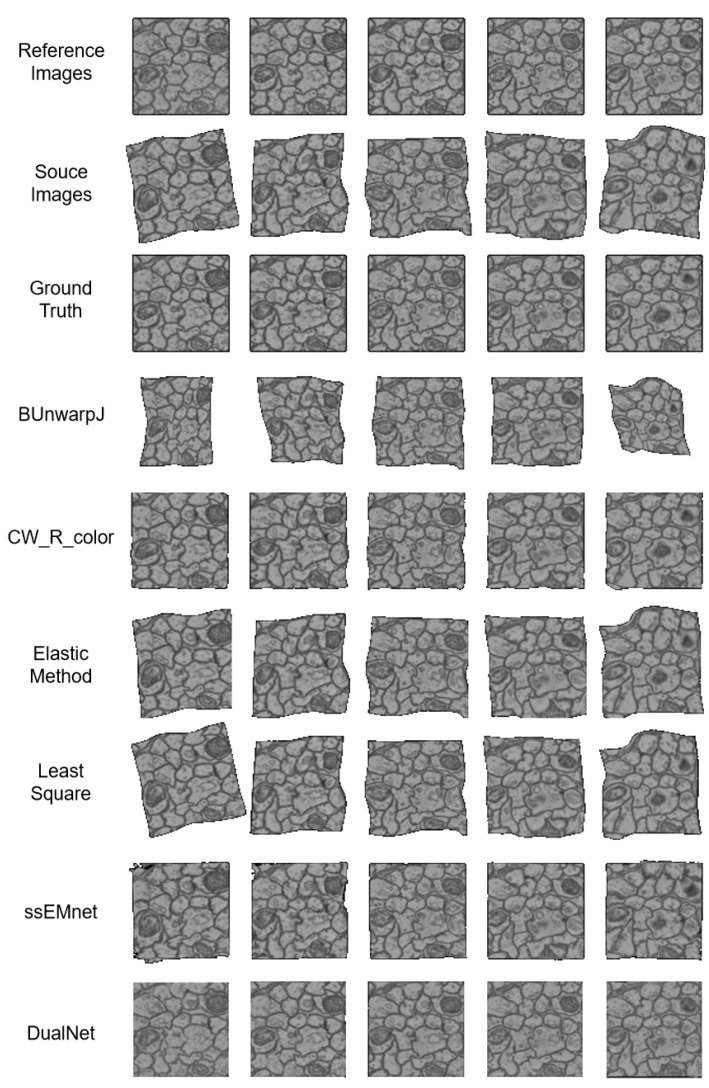
The registration results of baseline methods and our DualNet.

Some methods with publicly available codes, including the Elastic Method [11], LeastSquare [8], BUnwarpJ [9], CW_R_color [10], Pairwise [26] and ssEMnet [15], are used as baselines. Table 3 shows the comparison between our methods and baseline methods in terms of accuracy and speed. Figure 2 shows a qualitative study between our methods and baseline methods.

**Speed.** All the methods’ speeds are evaluated on the same equipment. Our DualNet method outperforms the other methods by a large margin. Traditional methods fail to catch up with the speed of deep learning-based methods (ssEMnet and DualNet), which tend to be 3–30-times faster. For traditional methods, their optimization processes have to run once per input. Their optimization is usually conducted in an iterative manner, which is time consuming and inefficient. In contrast, once deep learning-based methods have been trained, their models do not need to go through the complex optimization process during use.

Compared with the deep learning-based method ssEMnet, our DualNet is 7-times faster. Unlike DualNet, which uses grayscale images as inputs, ssEMnet is designed to have a substantially more complex architecture to receive feature maps extracted from original image pairs by a pretrained encoder. Its loss function is evaluated at the feature level, which means greater computational burden. In contrast, our proposed loss function is able to substitute the role of the feature-level comparison and will not cause excessive computational complexity.

**Accuracy.** In terms of accuracy, our method outperforms all the state-of-the-art methods. The first metric, SSIM, is aimed at measuring the continuity between registered image pairs. From this aspect, our method achieves substantially more continuous results. As shown in Figure 2, most methods fail to eliminate the deformation between the reference images and the source images. CW_R_color and ssEMnet remove most of the deformation but fail to obtain clear boundaries, in contrast to our method.

The second metric, Dice, is designed to determine whether the proposed method is able to restore the connectivity between mechanically cut neurons; this ability is of great significance in the neuroscience field. Furthermore, unlike the previous SSIM metric, Dice is measured with ground truth, meaning it is more likely to reveal a method’s capability for real-world applications. From this perspective, our method is more applicable than the other methods.

### 4.4. Analysis

**Figure 3:**
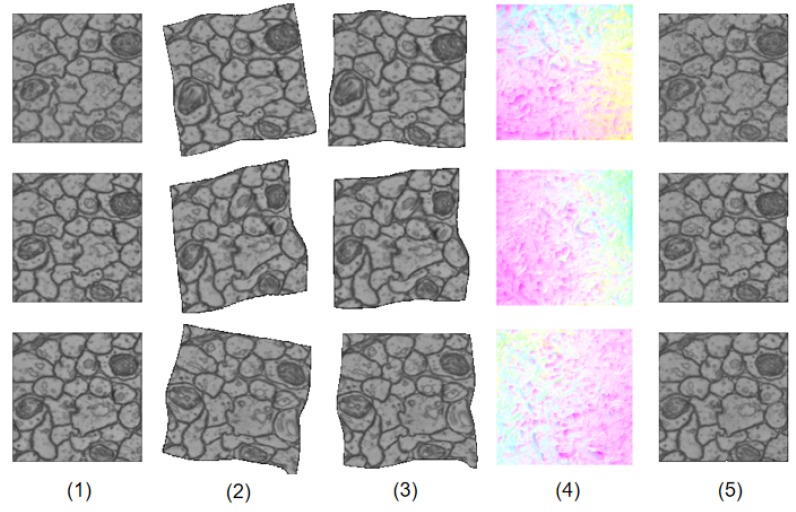
An illustration of how DualNet works. (**1**) reference images. (**2**) source images. (**3**) linearly transformed images. (**4**) nonlinear deformation filed. (**5**) final results.

Figure 3 shows how LinearNet and NonlinearNet work together to generate the final results. LinearNet outputs an affine transformation to linearly warp the original source images, resulting in the images in the third column. At this time, the linear deformation has been largely eliminated, resulting in linearly warped images becoming good initial estimations for subsequent nonlinear deformation regression. The nonlinear deformation field estimated by NonlinearNet is visualized in the fourth column. We can see that the nonlinear deformation field focuses on the local variations of fine details, which coincides with our original design principles. After two-stage warping, the final results are very similar to the reference images, as shown in the last column.

When removing NonlinearNet, it is obvious that the remaining LinearNet is not able to obtain satisfactory registered images, whereas when removing LinearNet, NonlinearNet will have trouble regressing the global transformation; when high-level semantic features are needed, the current simple architecture is not sufficient. However, the proposed divide-and-conquer scheme makes LinearNet and NonlinearNet excel in their advantages and make up for each other’s drawbacks, resulting in a simple but efficient architecture to accomplish the complex brain image registration task. Since the architectures of the two subnetworks are all simple, the overall dual-network architecture is also very simple, and it has much fewer learnable parameters than widely used architectures such as the flownet [12] and flownet2 [13] architectures.

During our experiments, we observed that when the linear deformation estimation fails to obtain correct results, the subsequent nonlinear deformation estimation usually cannot provide promising results. Once the linear deformation has been well estimated, the further nonlinear deformation estimation becomes very easy. Based on this phenomenon, we can conclude that introducing a linear deformation estimation step will dramatically simplify the remaining nonlinear deformation estimation, and directly estimating a nonlinear deformation is very difficult.

### 4.5. Ablation Study

To better understand how different elements influence the overall performance, in Table 4 and Table 5, we perform an ablation study by changing the settings of our model.

**Training Loss.** To clarify how much each component of the overall loss contributes to the overall performance, we try different combinations of losses, as shown in Table 4.

We can see from Table 4 that the SSIM loss is of the greatest importance and contributes to the largest performance boost. This is because the SSIM metric is a sensitive metric that can distinguish differences at small details. We experimentally found that when $L_{I}$ decreases to near zero, $L_{SSIM}$ still has a relatively high value and is still able to guide the training of the dual network. The two constraints are also indispensable; they provide appropriate regularizations for two types of deformation fields, preventing the estimation of unreasonable deformation fields. Without these two constraints, the dual network will require far longer to converge since a random initialization sometimes leads to a bad starting point for optimization, and it will take a long time for the network to return to a good state. The introduction of these two terms provides the correct optimization direction regardless of whether the network is well initialized.

**Image resolution.** Since in our setting the input image resolution has a large impact on the final performance, to determine the configuration of th input image resolution that will lead to the best performance with as short a computation time as possible, we test different combinations of input image resolutions in LinearNet and NonlinearNet, as illustrated in Table 5.

By comparing the figures in the top half and bottom half of Table 5, we can conclude that reducing the image resolution of the NonlinearNet inputs will lead to a dramatic decrease in the overall accuracy. Because the nonlinear deformation is very sensitive to local details, the image resolution becomes very important for its regression. Therefore, for NonlinearNet, we should also provide as many high-resolution inputs as possible.

From the results in the first three rows, we can conclude that the increase in input image resolution may not necessarily improve the performance of LinearNet. The most appropriate image resolution for LinearNet is 256×256, and higher or lower resolutions will all lead to a loss of accuracy. Since LinearNet has a simple architecture, an excessive image resolution will result in its receptive field being exceeded; under this circumstance, LinearNet cannot extract all the global information, and a loss of accuracy is inevitable. Too low of an image resolution will not provide sufficiently clear structural information, and this phenomenon will be magnified by the downsampling operations of LinearNet; the overall accuracy will also decrease.

Even though reducing the input image resolution will reduce the time required for prediction, our network has a simple architecture, it is sufficiently fast, and the difference between different image resolutions is not substantial; thus, we can choose a relatively high solution — 256×256.

## 5. Conclusions

In this work, we aim to solve the image registration problem, which is a key component of volume reconstruction from brain microscopy sections. In contrast to common methods that design complex models to regress the deformation between two given images, we suggest that complex models are unnecessary and propose a dual network with a simple architecture to solve this problem. We divide the original deformation field estimation problem into two subproblems. The two branches of the dual network fully utilize the individual properties of these subproblems and solve them in a simple yet efficient manner. To make our method applicable to real-world scenarios, we propose a series of loss functions to enable unsupervised learning, and no ground truth is needed during training. Experiments on fly brain electron microscopy images show that our method achieves the best performance with the lowest time requirement. Comprehensive ablation studies further verify the effectiveness of our work. In this work, simple convolution layers are used, some modules which have been proven extremely computation-efficient, e.g., EfficientNet [27], ShuffleNet [28] and MobileNet [29] can also be adopted in our architecture, we will focus on efficiency in our future work. 

## Figures and Tables

**Table 1:** **LinearNet architecture.** Here k is the kernel size, s is the stride, chns is the number of output channels for each layer, res is the input image resolution, and input corresponds to the input of each layer, conv* denotes convolutional layer.

LinearNet						
**Layer**	**k**	**s**	**chns**	**res**	**Input**	**Activation**
conv1	7	2	64	128×128	images	ReLU
conv2	3	2	256	64×64	conv1	ReLU
conv3	3	2	512	32×32	conv2	ReLU
conv4	3	2	512	16×16	conv3	ReLU
conv5	3	2	512	8×8	conv4	ReLU
conv6	3	1	256	8×8	conv5	-
conv7	3	1	64	8×8	conv6	-
conv8	3	1	6	8×8	conv7	-
GAP	8	1	6	8×8	conv8	-

**Table 2:** **NonlinearNet architecture.** Here k is the kernel size, s is the stride, chns is the number of output channels for each layer, res is the input image resolution, and input corresponds to the input of each layer where ↑ is a 2× nearest-neighbor upsampling of the layer, conv* denotes convolutional layer, deconv* denotes transpose convolutional layer.

NonlinearNet						
**Layer**	**k**	**s**	**chns**	**res**	**Input**	**Activation**
conv1	7	2	64	512×512	images	BN+LeakyReLU
conv2	3	2	128	256×256	conv1	BN+LeakyReLU
conv3	3	2	256	128×128	conv2	BN+LeakyReLU
conv4	3	2	512	64×64	conv3	BN+LeakyReLU
conv5	3	1	256	64×64	↑conv4+conv3	BN+LeakyReLU
conv6	3	1	128	128×128	↑conv5+conv2	BN+LeakyReLU
conv7	3	1	64	256×256	↑conv6+conv1	BN+LeakyReLU
conv8	3	1	2	512×512	↑conv7	Tanh

**Table 3:** Quantitative results comparison on fly brain image data.

Method	SSIM	Dice	Time (s)
Elastic Method [11]	0.587	0.764	1.654
LeastSquare [8]	0.591	0.710	3.019
BUnwarpJ [9]	0.606	0.652	2.259
CW_R_color [10]	0.646	-	2.395
Pairwise [26]	0.464	0.575	2.225
ssEMnet [15]	0.688	0.782	0.733
DualNet	0.758	0.812	0.104

**Table 4:** Ablation study on varying loss combination.

Loss	SSIM	Dice	Time (s)
$L_{I}$	0.711	0.775	0.101
$L_{I}+L_{SSIM}$	0.732	0.794	0.103
$L_{I}+L_{SSIM}+L_{lc}$	0.746	0.805	0.103
$L_{I}+L_{SSIM}+L_{lc}+L_{nc}$	0.758	0.812	0.104

**Table 5:** Ablation study on varying input image resolutions.

LinearNet	Nonlinearnet	SSIM	Dice	Time (s)
512×512	512×512	0.741	0.797	0.135
256×256	512×512	0.758	0.812	0.104
128×128	512×512	0.742	0.797	0.082
64×64	512×512	0.739	0.785	0.067
512×512	256×256	0.711	0.768	0.109
256×256	256×256	0.708	0.779	0.081
128×128	256×256	0.706	0.759	0.067
64×64	256×256	0.695	0.763	0.051
